# The Camp of Reason: Spinoza's *Ethics* as Affirmative Excess

**DOI:** 10.1111/nup.70081

**Published:** 2026-03-30

**Authors:** Jamie Brian Smith

**Affiliations:** ^1^ Gender in Medicine, Charité – Universitätsmedizin Berlin Germany

**Keywords:** affect, affirmative ethics, camp, Euclidean drag, *laetitia*, nursing philosophy, Spinoza

## Abstract

Perhaps Spinoza's *Ethics* is the most austere text in Western philosophy: axioms, definitions, propositions, demonstrations, arranged with the composure of a Euclidean proof. Yet readers who persist report something closer to exhilaration than to the satisfaction of verification. This paper asks why. Drawing on Sontag and Cleto, I argue that camp (not subcultural taste but stylised excess performed with affectionate seriousness) names a structural property of the geometric method itself. The *more geometrico* is so committed to deductive rigour that the commitment becomes performative, generating the very *laetitia* (joy as the passage to greater power to act) it theorises. I call this device Euclidean drag: drag as an inhabited role whose apparatus becomes luminous, and as the accumulated pull of proof drawing the reader into expanded capacity. The reading offers an account of reason as joyful, connective, and non‐moralising. Drawing on Sedgwick's reparative reading, I argue that camp's political force (converting what excludes into what sustains) survives this extension because the structural operation, not the subcultural origin, does the work. The account is consequential for nursing and for any practice whose practitioners inhabit formal protocols under conditions where structure might invite competence rather than compel compliance, and for what this special issue names the politics of seriousness.

I shall consider human actions and appetites just as if it were a question of lines, planes, and bodies.

— Spinoza, Ethics III Preface

## 1 A Puzzle About Reason

Consider that The *Ethics* is the most absurd, obtuse and severe text in Western philosophy. It derives an infinite being from three modest axioms, diagrams jealousy with laser precision, and submits the passions (desire, love, hatred, hope, remorse) to the apparatus of mathematical proofs, as though grief were so many intersecting planes. Allon White (1993, p. 124) called Reason the “dismal sacred word” of academic discourse, and anything written in the geometric manner of Euclid ought, by rights, to be the dreariest of that dreariness: Reason squared, Reason without remainder, Reason polished until nothing but Reason is left. The *Ethics* defeats this expectation.

Readers who stay with the proofs, work through the notes, and let the connections build up often find something closer to exhilaration than simple confirmation (Lloyd 1996, pp. 62–64). The joy is not separate from the rigour but produced by it. The spare, formal machinery creates feeling, and what it creates is something more frustrating, lighter, stranger than the satisfaction of mastery — perhaps closer to affirmation. This raises a puzzle about reason. It cannot be resolved by pointing to Spinoza's conclusions as his account of freedom, his refusal of moral judgement. The joy arrives before the reader gets there. It arrives in the working‐through, in the experience of following proofs whose steady composure never breaks. How can a text so committed to formal austerity, so stripped of ornament, personality, and rhetorical warmth, produce joy in its readers? Reason arrives in the *Ethics* doing more than it promises by exceeding the adequate and tipping into something extravagant, something camp.

In this paper, I make two claims. The first: camp names a style of reasoning that converts shame into recognition and capacities (Sontag 1966; Cleto 1999). The second: Spinoza's *Ethics* is already camp in form. Its more geometrico (defined in the following section) is so committed to rigour that the commitment becomes performative, generating the very *laetitia* it theorises. I call this device Euclidean drag. The discipline where this reading arrives is nursing, which has its own geometric manner — clinical protocols, assessment frameworks, evidence‐based guidelines whose formal severity rivals the *more geometrico* — and its own politics of seriousness in which professional knowledge must arrive stripped of affect to count as knowledge at all (Holmes and Gastaldo 2002; Purkis and Bjornsdottir 2006). If Spinoza's rationalism generates joy rather than compliance, the consequences for how nursing enacts and transmits its knowledge are direct.

## 2 A Note on Terms (Or: What Spinoza Means, Not What You Think He Means)

The *Ethics* has its own vocabulary, and much of it collides with everyday usage. The following terms appear throughout this paper in Spinoza's sense, which is often not the obvious one.

### 2.1 God

Substance itself. The single infinite reality of which everything that exists is an expression. Spinoza was excommunicated for this.

### 2.2 Substance

That which exists in itself and is conceived through itself. There is only one of it, and it is everything.

### 2.3 Affect

Any change in the body's capacity to act and think. Joy, sadness, desire — these are affects. So is the feeling of a proof clicking into place.

### 2.4 Laetitia

Joy, understood specifically as the body's passage to greater power of acting. Augmentation as a process rather than happiness as a mood.

### 2.5 Hilaritas

Cheerfulness. A form of *laetitia* that strengthens the whole body rather than any single part. The closest Spinoza gets to describing what it feels like to understand something properly.

### 2.6 Adequate Idea

A thought that carries its own evidence. You know you understand because the understanding works, because it connects to other ideas and extends them.

### 2.7 More Geometrico

“In the geometric manner.” Spinoza's method of writing philosophy as though it were Euclid: definitions, axioms, propositions, demonstrations. The method this paper argues is camp.

### 2.8 Camp

Stylised excess performed with affectionate seriousness. The sensibility that takes form so seriously the form becomes visible as form, and in doing so creates shared competence rather than hierarchy. A clinical protocol performed with enough care that the practitioner grasps its reasoning is closer to camp than to compliance.

### 2.9 Euclidean Drag

This paper's term. Drag as inhabiting a role so thoroughly the apparatus becomes its own spectacle; drag as the accumulated pull of proof drawing the reader forward. The method is so straight it bends.

## 3 Camp, Precisely

Sontag's “Notes on ‘Camp’” (1966) remains my starting point^1^ because it names the phenomenon with enough precision to be philosophically useful: camp is stylised excess performed with affectionate seriousness, or the sensibility “serious about the frivolous, frivolous about the serious” (1966, p. 288). What Sontag documents is a posture toward form, an elaboration that exceeds the occasion, and refuses to apologise for the excess. Her account has known limitations: it leans toward a taxonomy of taste, a catalogue of objects that are camp (Tiffany lamps, Flash Gordon, certain operas), risking reduction to a sensibility possessed by certain readers when camp is better understood as a property performed by certain texts (Cleto 1999, p. 3). Cleto relocates camp from the eye of the beholder to the performed relation between text and audience: camp is something a text does, not something a reader is and as such describes camp as an excess without apology enacted at the level of form (Cleto 1999, p. 3). Newton (1972) ethnography of drag performers documents the operation at the level of embodied practice: the queens she studied inhabited femininity with such commitment that the role became visible as role, yielding not mockery but shared facility, a competence that penetrated and pinned the audience. Camp is recognisable by its effects: the recognition laugh that passes through a room, the shared facility that follows, the quiet conversion of embarrassment into belonging.

Camp must be distinguished from its close neighbours, and the distinctions are where its epistemological force becomes apparent.^2^ Where irony distances the speaker from the material, establishes superiority by breaking the straight face with a wink, and its effect is a sense of being cleverer than the thing observed. Camp draws close, sustains the straight face beyond the point where irony would crack, and invites belonging. Where kitsch is naive excess, sincerity that has not yet examined itself, begging for sentimentality not yet interrogated by logic(s). Camp is knowing excess and an excess of knowing, celebrating form as form where kitsch might ignore form altogether. Where parody targets its object, creating possibilities to diminish through exaggeration, and the laugh is directed at someone; in camp, nobody is the butt, and the exaggeration makes the form available for shared use. The discriminator is directionality because irony, kitsch, and parody create asymmetry; camp creates multidimensional, polygonal, recognition. The camp performer holding the straight face beyond necessity is doing pedagogical work and the audience recognises the form as form, and in that recognition acquires the facility to handle it, extend it, pass it on. Camp can convert the anxious do I belong? into the pleasurable I see what you are doing, and I can do it too.

Sedgwick (2003, pp. 123–152) account of reparative reading sharpens the claim. Where paranoid reading, her term for the hermeneutics of suspicion, arms itself against surprise by anticipating the worst, reparative reading allows itself to be nourished by its objects, assembling resources for sustenance rather than evidence for prosecution. Camp, on Sedgwick's account, is reparative reading's characteristic mode: it takes pleasure in the textures of form because form can be inhabited, shared, and passed on (see also Halperin 2012, pp. 183–227, on camp as a mode of cultural transmission). Muñoz (1999, pp. 11–34) presses the point into the register of survival, documenting how disidentification — working on and against dominant cultural forms rather than assimilating or rejecting them — enables subjects positioned outside the mainstream to build liveable worlds from unpromising materials. Camp's political force is the conversion of what excludes into what sustains. Whether a seventeenth‐century rationalist text can perform the same operation, and whether it matters for practitioners who work within formal structures they did not design, is the question.

Genevieve Lloyd's reading of the *Ethics* provides the affirmative counterpart to camp's structural account.^3^ Lloyd argues that the *Ethics* enacts an affirmative account of human flourishing grounded in the body's increased capacity to act, beyond moral prohibition (Lloyd 1996, pp. 67–72; Lloyd 1994). Spinoza's term is *laetitia*; its fullest expression is *hilaritas*. Gatens and Lloyd (1999) take this further into relational territory: adequate ideas are compositional, grow with experience and encounter, and are irreducibly social (Gatens and Lloyd 1999, pp. 24–31).

Gilles Deleuze makes a complementary move: for him, Spinoza's ethics is an ethology, a study of what bodies can do. This becomes a story of capacities; what bodies have the capacity to do, how and why their capacities to affect and be affected change. On this reading, the passions are affects to be understood, composed, and where possible strengthened (Deleuze 1988, pp. 17–29). Deleuze's earlier study of Spinoza (1968/1990) develops an account of expression that bears on my argument: substance expresses itself through its attributes, and the expression is not a representation of something hidden but is itself the reality. The geometric method, on this account, does not describe the structure of things; it participates in it. What Deleuze's readings open up is a shift in attention from what the *Ethics* says to what it makes possible (see also Sharp 2011; Montag 1999). Yet what the proofs do to the reader is what interests me here. Not only what the text argues about affects but what affects it generates in those who work through it, and whether that generation is incidental to the philosophy or constitutive of it.

## 4 The *Ethics* As Euclidean Drag

Spinoza announces his intentions in Part III's Preface (the epigraph to this paper) with a sobriety that borders on provocation in that he will treat human affects (desire, love, hatred, jealousy, the passions philosophy had for centuries treated as moral failings or divine punishment) “just as if it were a question of lines, planes, and bodies” (Spinoza 1996, *Ethics* III Preface). What makes it camp, in Cleto's sense, is what Spinoza does next: he acknowledges what other philosophers would have found absurd and presses ahead with undiminished commitment. This is the camp gesture Cleto (1999, p. 3) identifies: excess without apology, the acknowledgement of one's own outrageousness followed by redoubled conviction. The *more geometrico* is promised, nodded to and enacted, less a warning than an invitation to enjoy the ride. Definitions stake claims about what things are; axioms declare what needs no proof; propositions build from these materials; demonstrations exhibit the scaffolding; corollaries extend the reach; and scholia step aside to comment, recontextualise, or land a point the deductive architecture cannot accommodate. The whole thing rises, each element supporting the next, outstripping any reader's ability to hold it in view.

The scholia of The *Ethics* deserve separate attention.^4^ They are where the double register becomes most audible. In a scholium, Spinoza addresses the reader directly. He steps out of the deductive architecture to remark on what has just been proved, forestall misreadings, settle scores with Descartes, or land an observation the geometric architecture cannot deliver on its own. The scholia are the stage directions of the text: they tell the reader how to receive what is unfolding, where to look, what the apparatus is doing and why. Lachterman (1989) argued that the geometric method was never a neutral container for ideas but always shaped what could be thought within it. In Spinoza's hands, the shaping becomes the teaching, and the *Ethics* becomes a camp classic. Consider the Scholium to Part III, Proposition 2. Spinoza interrupts a demonstration about mind and body to observe, almost parenthetically, that “no one has yet determined what the body can do” (Spinoza 1996, *Ethics* III P2 Schol.). It exploits the opening in the Cartesian framework where mind commands and body obeys, replacing command with an open question about corporeal power that seventeenth‐century philosophy had no apparatus to answer. But Spinoza delivers it as an aside, a remark nested in a scholium to a proposition about something else, with the composure of someone noting the weather. The gap between the modesty of delivery and the magnitude of what is being said is the teaching. The reader registers the claim and registers that it has been slipped in under the geometric radar; this double registration creates the attention the work demands.

Consider Proposition 11 of Part I, where the camp device is most immodest to his contemporaries. Spinoza's God is not the God of theology, not a creator who stands outside the world and judges it. God, as the *Ethics* defines the term, is substance itself: the single, infinite reality of which everything that exists is an expression. The move scandalised Spinoza and saw him excommunicated from his synagogue; it remains disorienting for some now. Having redefined God in this way, Spinoza provides not one but three demonstrations of God's existence, each departing from a different starting point, as though once were not enough, or more precisely, as though the proposition deserved the full treatment.

The first proof works by contradiction: substance's non‐existence contradicts its own definition, and the assumption collapses within a few steps (Spinoza 1996, *Ethics* I P7; *Ethics* I P11 Dem.). The second approaches from causal reasoning: for anything that exists or does not exist, there must be a reason or cause, and nothing outside substance could prevent it from existing because nothing outside substance exists (*Ethics* I P11 Alt. Dem. 1). The third appeals to power: the ability to exist is itself a capacity, and if only finite things existed the finite would have more power than the infinite, which Spinoza calls “absurd” (*Ethics* I P11 Alt. Dem. 2). Whether any individual proof convinces depends on whether the reader accepts the definitions Spinoza laid down at the outset; the definitions are not argued for but declared, and the declaration is part of the performance. What matters for my argument is the accumulation. Three proofs, three routes to the same destination, each delivered with the same geometric composure. The insistence is itself part of the device; it produces conviction through stubborn architectural commitment (see Curley 1988, pp. 9–12, on the relation between the method and the argument's force).

The premises are deceptively plain, and some would say as clear as mud. From these unpromising starting points Spinoza derives a claim that most of his contemporaries would not have dared to write down: that everything that exists is an expression of one substance, and that this substance could not have been otherwise (*Ethics* I P14, P15). He committed it to paper, in geometric form, and published it. That act of commitment is where the camp lives. The distance between the composed gesture and its consequence, the deadpan delivery that refuses to acknowledge the scale of what it has just done, and done in the most wilfully obtuse way possible; this is the mismatch. The vexation is the teaching. You grasp the proof, though you are never entirely sure of your grasp, because what you have hold of is not a settled conclusion but a continuing chain of deduction about the nature of material reality. And you grasp that the proof is extraordinary, and the two arriving together, the uncertain hold and the extraordinary reach, is what produces the pleasure. Look how much can follow from so little, provided you follow carefully. And the reader, having followed carefully, finds they have followed further than they expected to go.

The distinction between this experience and the mathematical sublime is precise. The sublime overwhelms when you encounter something so vast it makes you feel small, and the pleasure comes from the smallness, from being awed by what you cannot contain. Camp recognition works the other way round. The reader who follows Spinoza's derivation of an infinite being from three axioms does not feel diminished; they have followed each step, grasping how it works, and could reconstruct it, teach it to someone else, take it further. The joy is a passage to greater power, not a recognition of powerlessness. The sublime says: how vast. Camp says: look how much we can do with this. The same device operates at the other end of the affective scale. In Part III, Proposition 35, Scholium, Spinoza treats jealousy as a geometric theorem (Spinoza 1996, *Ethics* III P35 Schol.). Your neighbour acquires the thing you have coveted; Spinoza traces the feeling step by step, from the moment you see someone else wanting what you want, through the sadness of imagining you have lost it, to the hatred that settles on the person who has it. He diagrams jealousy the way an engineer might diagram a bridge. Sontag (1966, p. 287) wrote that camp is “the sensibility of failed seriousness, of the theatricalization of experience”; she might equally have noted its inverse, the sensibility of seriousness so total that it theatricalises itself. The precision applied to jealousy is absurd; the absurdity is precise. To read it is to experience both at once, and the experience is not mockery but recognition.

I call this device Euclidean drag.^5^ The word carries a double register. Drag is, first, enactment of a role so committed that the commitment reveals the role as role. More than parody, which mocks its object, but inhabiting so thoroughly that the apparatus becomes its own spectacle (Cleto 1999, p. 18). Drag is, second, the accumulated pull of a long text: proof upon proof, scholia as stage directions, cross‐references as choreography, each proposition both freestanding and dependent on everything that came before. Allon White's phrase for what academic discourse made of reason, the “dismal sacred word” (White 1993, p. 124), names what Spinoza refuses. On Spinoza's own epistemology, whether Euclidean drag belongs to the text or to the reader is a distinction without a difference: adequate ideas are relational, produced in the encounter between bodies, and the text's camp is realised in the reading as the reading is shaped by the text (*Ethics* II P13–P16). The method is so straight it bends.

What happens when you read the *Ethics* from Part I through Part V in one sitting? The accumulation creates a logical vertigo. By the time you reach Part V, On the Power of the Intellect, or On Human Freedom, you cannot hold the book in view. The chain of demonstrations stretches behind you like a proof that has outgrown your ability to survey it, and the text keeps moving. It is an endurance sport. The work performs a double gesture while it demonstrates that everything is in principle knowable (every affect derivable, every relation traceable) while demonstrating that no finite mind can know it all, because substance expresses itself in infinite attributes and the world is always in motion (Spinoza 1996, *Ethics* II P7 Schol.; Deleuze 1968/1990). Knowledge, the text teaches by enacting, must be situated and relational, never omniscient. Each demonstration extends or diffracts what you can think; each cross‐reference sends you back through earlier proofs, and the retracing deepens without repeating. Parts III and IV are where the choreography becomes most demanding. The affective taxonomy thickens, the cross‐references multiply, and the reader finds herself holding three or four threads simultaneously, unable to reduce any of them to a paraphrase that would release them from the form. The text demands patience; you learn to dwell in the proof, to let the architecture carry you instead of racing toward the conclusion. The feeling of the argument clicking into place is the reading itself, registered as augmentation.

What I call the mask of deduction is this: the geometric format presents itself as a transparent vehicle for conclusions, as though the method were merely a window onto the content (Lloyd 1996, pp. 40–44, 62–64). The presentation is itself a performance; its composure, its refusal to raise its voice, the care visible in every cross‐reference shape how readers feel about the material. The mask is the face, because there is nothing behind the geometric method except the geometric method. The rigour is the warmth; the deduction is the invitation.

Spinoza scholarship has long debated whether the geometric method is merely a way of presenting ideas that could be expressed differently, or whether the form itself shapes what the ideas can mean (Curley 1988, pp. 9–12; Gueroult 1968). Curley argues that Spinoza's arguments can be pulled out of their geometric housing and restated in ordinary prose without losing their logical force. On this reading, the proofs are what matter; the geometric format is a container, useful but not essential. Gueroult argues the opposite: that the order of demonstration is inseparable from what is being demonstrated, and the propositions could not mean what they mean outside the architecture that creates them. The camp reading aligns with Gueroult's position and extends it into affective territory neither he nor Curley addressed: the method generates the content and the reader's felt relationship to that content simultaneously. This changes what we think the geometric method is for. If the method is just a container for conclusions, then whatever the reader feels along the way is incidental, a by‐product of clear presentation. If the method is constitutive, the joy is philosophical: it is produced by the form itself, and is part of what the *Ethics* teaches. The joy need not be pleasant in any straightforward sense. Working through the *Ethics* is closer to running a marathon than to reading a well‐turned sentence; the satisfaction arrives through difficulty, not despite it. But the satisfaction is not incidental to the method, it is the method doing what it was built to do.

Part I opens with eight definitions and seven axioms before a single proposition appears, and the definitions do not invite discussion: “By substance I understand what is in itself and is conceived through itself” (Spinoza 1996, *Ethics* I Def. 3). The reader who does not already know the philosophical vocabularies of Spinoza will feel the definitions as demands. Then understanding arrives. The proof lands; what seemed opaque becomes clear and usable; you can extend it, teach it to someone else. The affective conversion, the shift from frustration to capacity or facility, maps onto Spinoza's own taxonomy. This creates a circularity, since the framework analysing the reading experience is the same framework the reading produces, but one Spinoza's epistemology anticipates (*Ethics* II P43). The reader who grasps a proof and feels the click of comprehension is already experiencing what the text describes.

The conversions are specific. Shame — “sadness accompanied by the idea of some action which we imagine others blame” (*Ethics* III P30 Schol.) — is a feeling most readers will recognise in not understanding what everyone else seems to grasp: the student who nods along while lost, the practitioner who follows the protocol without grasping the reasoning. In the reading, that shame becomes curiosity, displacing the passive fear of being found out. Fear of not following is another: the anxiety of looking ahead and expecting to fail, I will not understand Part III if I cannot hold Part I. That fear becomes foresight, the ability to anticipate where the argument is heading before it arrives; foresight grows from understanding rather than guesswork, and accompanies the body's passage to greater power. Confusion is the third: partial ideas that interfere with one another (*Ethics* II P29 Schol.), threads tangling. That confusion becomes clear perception of how those ideas relate; the web of cross‐references that bewildered the reader now organises their thought. But the relief is temporary; each resolution opens onto the next opportunity for deduction, and there is always more to understand. The text offers an ongoing practice of converting confusion into comprehension, one proof at a time, knowing the chain continues.

Each conversion follows the same pattern. A passive affect, one whose cause lies outside the reader's understanding, is replaced by an active one, whose cause the reader now comprehends. The replacement does not simply swap one feeling for another; it transforms the feeling's quality. The recognition laugh, the quiet “ah, yes” that accompanies comprehension clicking into place, is reasoning's affective signature. Hilaritas is not a reward for understanding; it is understanding's own texture.

The recognition is not private. Reading the *Ethics* has characteristically been collective: the intellectual circles of seventeenth‐century Amsterdam where Spinoza's text circulated in manuscript; the reading groups that have persisted in philosophy departments and self‐organised collectives since; the tradition of Spinoza scholarship, which proceeds less by refutation than by communal re‐reading (Gatens and Lloyd 1999, pp. 1–12). This paper was planned after a communal reading. A proof that outstrips any individual's ability to hold it in view invites collective engagement; someone holds the thread of Part I while another traces the affect through Part III, and the joint reading yields understanding that neither practitioner would reach alone. The work is structurally sociable. Gatens and Lloyd (1999, p. 143) named this “collective imaginings”: ways of understanding that emerge through thinking together. Euclidean drag specifies how such imaginings form.

The device operates across the whole text. Part V, On the Power of the Intellect, opens by announcing its subject as “the way or method leading to freedom” (Spinoza 1996, *Ethics* V Preface), then delivers it through the same geometric apparatus that diagrammed jealousy in Part III. Proposition 23 demonstrates that the mind cannot be entirely destroyed with the body; Proposition 36 concludes that the intellectual love of God is eternal. These are not modest claims, and the geometric composure with which they arrive is itself the teaching: the most extraordinary conclusions are delivered with the same architectural discipline as the most mundane definitions. The camp device does not flag or diminish across the five parts; it intensifies. Any practice whose practitioners inhabit formal structures under pressure will recognise the operation.

## 5 Objections and Replies

The *Ethics* conducts its most candid self‐examination in its scholia, where objections are raised and met within the architecture of the proof rather than appended after it. What follows takes a nod from Baruch: five objections a careful reader might raise, with replies that specify what survives the challenge.

### 5.1 Objection 1

The camp reading trivialises Spinoza. To call the *Ethics* “camp” is to reduce a serious philosophical achievement to an aesthetic category better suited elsewhere.

### 5.2 Reply 1

Camp, as defined here following Cleto (1999, p. 3), is not ridicule. It names a feature of texts that teach by making their form visible through stylised excess. To identify the *more geometrico* as camp is not to place the *Ethics* alongside Flash Gordon but to specify how its geometric method continues to create competent readers. The trivialisation charge assumes camp diminishes its object; the reading extends Lloyd (1996, pp. 40–44) claim that the *Ethics* teaches through form as much as content. I am specifying what that pedagogy feels like, and the feeling is part of the philosophical work.

### 5.3 Objection 2

Sontag's camp is apolitical. Borrowing her framework aestheticises what should be a question of power.

### 5.4 Reply 2

The objection would land if I had adopted Sontag (1966) account wholesale; I have not. Nothing is apolitical. The test I propose is Spinozan rather than Sontagian, which is to say, does the performance increase shared power to act, or does it diminish it? This criterion is political in the sense that matters; it asks who gains capacity and who loses it when a proof, a pedagogy, or a protocol is enacted. Camp‐reason converts shame into collective capacity; a performance that converted competence into individual prestige would fail the test. The Spinozan criterion supplies what Sontag's aestheticism lacks: a measure grounded in whether bodies' capacities to act and think together are augmented or diminished.

### 5.5 Objection 3

The *Ethics* is austere, not excessive. The geometric method strips away ornament; it is the opposite of camp's theatrical surplus.

### 5.6 Reply 3

Austerity and excess are not opposites when the austerity is so unbroken, so unblinking, and brutal that it becomes its own spectacle. A few lines of deductive proof yield a never‐ending, eternal substance; human jealousy is diagrammed with atomic precision. The geometric method applied to the passions is not austere so much as flamboyantly austere, austere past the point where austerity remains simply itself.

### 5.7 Objection 4

Laughter is irrational. Introducing the “recognition laugh” into an account of Spinoza's rationalism undermines the rationalist reading from within.

### 5.8 Reply 4

Spinoza disagrees. As the account of *hilaritas* would suggest (*Ethics* IV P42; see Section 3), cheerful affects accompany the body's passage to greater perfection, which is what adequate understanding is on Spinoza's account. The recognition laugh (the quiet “ah, yes” when the proof clicks) is reasoning's affective signature. A genuine limitation must be acknowledged here because not every reader experiences this, and truthfully as an author, these moments are fleeting for me as I tussle with the brutalism of the text. Many find the *Ethics* alienating, impenetrable, or simply dull. If the camp reading depends on a particular kind of reader, as in one already equipped with philosophical patience and a disposition toward delight in formal systems, then Euclidean drag names a patient affordance of the text rather than an intrinsic property. I accept this. An affordance that reliably creates competent, joyful readers across four centuries of collective reading is philosophically consequential whether or not it operates universally. To treat laughter as the enemy of reason is to reproduce the moralist separation of intellect from affect that the *Ethics* dismantles.

### 5.9 Objection 5

Camp is a twentieth‐century queer subcultural phenomenon. Applying it to a seventeenth‐century rationalist text by a canonical philosopher risks gentrifying camp — evacuating its political force by extending it to a figure who needs no further celebration.

### 5.10 Reply 5

The gentrification risk is real, and I do not want to minimise it. Camp emerged from queer subcultural practice under conditions of exclusion; its political work was survival work, converting stigma into shared competence among communities for whom dominant forms offered only abjection (Newton 1972; Muñoz 1999). Applying camp to a canonical text could domesticate precisely what makes camp dangerous: its capacity to reorganise the relation between margin and centre. But camp names an operation, not an identity. The extension is legitimate only if three conditions hold: the structural operation (stylised excess making form visible as form) is present in the new context; the political test survives (shared capacity for whom? at whose expense?); and the reading acknowledges its debt to the queer subcultural practice that made the operation visible in the first place (see Halberstam 2011, pp. 1–25, on queer failure and alternative knowledge practices). If camp‐reason operates even in the text that epitomises philosophical seriousness, then seriousness itself has always depended on the affective operations camp names. Queer subcultural practice made those operations visible; it did not invent them. What camp's queer history insists upon is that form is political: how knowledge is practised determines who can participate in it. This paper does not substitute philosophical camp for queer camp. Whether the structural kinship I trace does justice to both remains a question the paper opens rather than settles.

## 6 What the Reading Produces

Camp and Spinozan affirmative ethics share a gesture: where moralism says “you should not feel that,” Spinoza says “let us understand what that feeling does”; where taste‐policing says “that is too much,” camp says “watch how much can be done with too much.” Both refuse the diminishing move and convert the refusal into increased capacity (Braidotti 2014, p. 168). If the style of reasoning creates its affective consequences, the question is where else this operates, and what happens to practitioners who work within formal structures whose affective dimension goes unexamined.

Nursing is the discipline I have in mind, though the argument does not depend on it. Since its nineteenth‐century professionalisation, nursing has been subject to a politics of seriousness: the demand that professional authority arrive solemn, clinical knowledge arrive stripped of affect, and the gap between protocol and practitioner narrow to the point of disappearance (Siles González 1999). The longer history of care — culturally, religiously, and metaphysically embedded — did not impose this demand; medicine, and nursing's institutional subordination to it, did. It operates through clinical protocols, assessment frameworks, care pathways, and evidence‐based guidelines, each with axioms (clinical evidence), definitions (operational criteria), propositions (recommended interventions), and demonstrations (documented outcomes). Holmes and Gastaldo (2002) argued these function as governmentality; protocols operate as technologies of compliance, not neutral epistemological tools. Smith et al. (2023; 2023) press the argument further, showing that healthcare algorithmics produce their own governmentality through the if–then logic of evidence‐based pathways. Purkis and Bjornsdottir (2006), writing in this journal, argued that nursing knowledge is enacted through practice, not applied to it — a constitutive account, and one whose logic the reading developed here shares. Whose clinical judgement arrives as decision and whose as observation requiring confirmation? Whose protocol knowledge counts as expertise and whose as mere compliance? When nursing knowledge must arrive solemn to be taken seriously, the demand itself performs the subordination it claims merely to reflect. Smith et al. (2024) name this demand the ‘Vitruvian nurse’: an impossible normative ideal against which all nursing performance is measured and found wanting.

What Benner (1982) documents as the cognitive arc from novice to expert and Carper (1978) maps as epistemic pluralism (empirical, aesthetic, personal, ethical knowledge operating simultaneously), Spinoza would recognise as the passage from passive to active affect (see also Tejeda Gómez et al. 2019). Both accounts describe the transition from compliance to competence but stop short of the affective passage itself: the felt shift when a practitioner moves from following a protocol because she must to inhabiting it because she understands. Camp‐reason names that register; the register in which clinical competence is experienced, not an addition to it.

Consider the handover. A structured clinical handover (situation, background, assessment, recommendation) can be performed as a bureaucratic requirement, a box to tick before going home. The outgoing nurse recites the template; the incoming nurse transcribes it; the template is completed and the patient becomes a data set transferred between shifts. The same handover, enacted with enough care that the incoming nurse grasps the reasoning behind the selection — why these observations were weighted, what the assessment excluded and why, where the plan of care might need to bend — creates something different. The incoming nurse leaves not merely informed but equipped: able to extend the reasoning, adapt the plan, explain it to the patient, teach it to a student. The structure is identical; what it does to the practitioner is not. The first handover diminishes; it produces a nurse who knows what to do next. The second augments; it produces a nurse who understands why, and can therefore act when the protocol runs out. The difference is Spinozan: the passage from passive affect (receiving information whose cause remains external) to active affect (comprehending the reasoning and making it one's own). Or the pain scale. Administered as a number to document, it yields a data point. Administered with enough attention that the patient grasps why the question matters and how the number connects to the plan of care, it creates a relation in which the patient participates in the reasoning. The structure is identical; the affective consequence is not. I do not prescribe how this should operate in clinical settings; the recognition, if it arrives, arrives in practice. In each case, the resolution is attention: attention to the patient as a participant in reasoning rather than an object of assessment, attention to the relational encounter in which care occurs, and attention to the practitioner's own passage from compliance to comprehension. The Spinozan claim is that this attention is not an addition to clinical competence but its affective core (Giménez Lorente and Siles González 2026; Díaz del Pino 2021; Garcia Uribe 2020).

Nothing in this argument asks practitioners to reason their way into accepting structures that diminish them. Where a protocol encodes bias, forecloses clinical judgement, or reduces care to data transfer, the Spinozan criterion applies to the structure itself: does it augment shared capacity, or does it not? The reading opens several responses beyond inhabitation: rethinking the structure so that its formal logic serves clinical reasoning rather than replacing it; allowing the relational encounter to override institutional rigidity when the protocol itself has become the obstacle; or recognising that in some contexts no protocol is the more adequate response, because the attention care demands cannot be algorithmically prescribed (Smith et al. 2023). What matters is not that practitioners learn to love their protocols but that they can distinguish structures that augment from structures that diminish, and act accordingly.

A queer rationalism, then. “Queer” here names a structural operation derived from the material conditions of queer lives, which it cannot substitute for. The rationalist tradition from Descartes onward has characteristically treated reason as the faculty that masters the passions and achieves certainty through eliminating ambiguity (Lloyd 1984). Spinoza's rationalism understands the passions where this tradition would master them, traces and celebrates the body's difference and complexity through infinite attributes, and seeks ongoing capaciousness; a passage to greater power with no stopping point because substance expresses itself infinitely. What makes this specifically queer is the structural kinship between Spinoza's refusal and the queer refusal of strict binaries; both inhabit a dominant form with such thoroughness that the form's possibilities exceed what its gatekeepers intended. Butler (1990, pp. 136–141) argued that gender is constituted through repeated performative acts rather than expressed from an interior essence; camp identifies an analogous operation in form, where repeated stylised excess makes the form visible as form and available for reworking. The analogy is structural, not identical: gender performativity operates under conditions of compulsion that Spinoza's geometric method does not share, but the mechanism of inhabiting a structure so thoroughly that it becomes visible as production is common to both. Spinoza inhabits rationality so completely that rationality becomes joyful, connective, capacious. The nurse who inhabits a clinical protocol with enough understanding to grasp its reasoning, adapt its application, and teach it to a colleague is doing something structurally similar. The protocol stops being a constraint and becomes a capacity.

Not all of Spinoza is camp. Not all camp is ethical; it can aestheticise suffering, serve reactionary ends, convert what should be outrage into mere style. Camp‐reason fails when poise becomes performance for its own sake, when the composure creates admiration where it should create comprehension, when evidence‐based method yields compliance in the team and prestige in the individual. The failure looks, from the outside, like success; the protocol is followed, the apparatus is sustained, the composure holds. The consultant whose clinical presentation leaves the team awed but no more competent; the lecturer whose command elicits admiration without producing understanding; the guideline whose rigour is so forbidding that practitioners follow it by rote, never grasping the reasoning behind it. In each case, conviction creates hierarchy, and the audience leaves diminished. Intention is irrelevant; the test remains Spinozan: does the enactment increase shared capacity? A straight face that creates silence where there should be questioning has not performed camp‐reason but cruelty in the costume of a nurse's uniform.

## 7 A Queer Rationalism, Calmly Stated

The device has been operating since 1677; our vocabularies for reason simply had no room for delight. This is a Spinozan answer to a Spinozan question, and its circularity is deliberate. The circularity follows from Spinoza's epistemology: adequate ideas are self‐certifying (*Ethics* II P43), though the self‐certification thesis has its critics and the camp reading does not depend on the strongest version of it; it depends only on the weaker claim that adequate understanding is recognisable from within the practice that produces it, as any practitioner who has felt a clinical protocol click into sense will confirm. If the camp reading has worked, it has worked by doing what it describes; if it has not, no concluding summary will repair the deficit. The question it opens for nursing philosophy is whether we can attend to the affective dimension of method with the same rigour we bring to its epistemic dimension, and the *Ethics* suggests that the two dimensions are one. Reason has no need to apologise for its pleasure in its own workings; it needs only to enact that pleasure with enough poise that others can join in. If the camp device has a motto, it is this: look how much we can do with this. The Ethics has been demonstrating as much since 1677, calmly stated, and with the extravagance it deserves.

## Funding

The author has nothing to report.

## Ethics Statement

The author has nothing to report.

## Conflicts of Interest

The author declares no conflicts of interest.

## Data Availability

The author has nothing to report.

## Endnotes

1 I take Sontag rather than later camp theorists (Newton 1972; Halperin 2012; Muñoz 1999) as the primary reference because her formalism is closest to the Spinozan register this paper requires. Newton's *Mother Camp* grounds camp in drag performers' lived practice; Halperin and Muñoz extend it into identity practice and queer world‐making. Their emphasis is politically generative, but would open a line of argument this paper cannot sustain at this length. Something is lost in the formalisation: extracting camp's device from the subcultural, embodied, and precarious contexts in which it developed risks a sanitised version, camp without the queens, as it were. I accept this cost because the structural strand is what makes the Spinoza reading possible. The formalisation does not, however, depoliticise camp. It extends camp's political operation and the conversion of shame into shared competence which is beyond the queer subcultural contexts where it was first identified. The Spinozan criterion (does the enactment increase shared power to act?) ensures the political orientation survives the extraction, because the criterion is itself a power analysis: it asks who gains capacity and who is diminished. The debt to queer practice should be named, and I name it here.

2 The claim that camp is epistemological rather than aesthetic resonates with, but is not derived from, Sedgwick's (2003) account of reparative reading. Both describe modes of engagement that increase the reader's or audience's power; both refuse the hermeneutics of suspicion. I note the resonance without pursuing it, as the Spinozan framework provides its own account of what “increased capacity” means.

3 The reading developed here engages primarily with the Lloyd–Deleuze–Gatens strand of Spinoza scholarship. The “new Spinozism”, particularly Lordon's (2014) account of how institutions harness affects, and Lord's (2010) discussion of the readerly experience of Spinoza's proofs, approaches the affective and political dimensions from directions that resonate with the camp reading. A full engagement exceeds this paper's scope, but the connection is worth noting: what Lordon describes at the macro‐political level (affect as institutional cement), this paper identifies in the micro‐phenomenology of reading.

4 The Bergsonian objection that Spinoza over‐trusts the intellect, spatialising duration and mistaking the map for the territory (Bergson, 1907/1998, pp. 354–361) is well taken on its own terms. The camp reading does not dispute it so much as repurpose it: the over‐trust is the enactment, the excess is the pedagogy. Spinoza's method does not claim to capture duration; it inhabits rationality at such pitch that the inhabiting itself becomes a site of learning. The limitation, read campfully, is the gift; you know it exists but it is there for you to find out.

5 I use “performance” in a sense closer to Butler's (1990) constitutive performativity than to theatrical performance, though the argument trades on the resonance between the two. Butler's point is that certain acts do not express a prior identity but create the identity they appear to describe. The *more geometrico* enacts rationalism in a double register: it stages rationality (theatrical sense) and the staging creates the rational capacity it describes (constitutive sense). The camp reading names the point at which the one becomes the other.
